# Stable isotopic evidence of nitrogen sources and C4 metabolism driving the world’s largest macroalgal green tides in the Yellow Sea

**DOI:** 10.1038/s41598-018-35309-3

**Published:** 2018-11-28

**Authors:** Ivan Valiela, Dongyan Liu, Javier Lloret, Kelsey Chenoweth, Daniella Hanacek

**Affiliations:** 1 The Ecosystems Center, Marine Biological Laboratory, Woods Hole, Massachusetts, 02543 USA; 2 State Key Laboratory of Estuarine and Coastal Research, East China Normal University, Shanghai, China

## Abstract

During recent years, rapid seasonal growth of macroalgae covered extensive areas within the Yellow Sea, developing the world’s most spatially extensive “green tide”. The remarkably fast accumulation of macroalgal biomass is the joint result of high nitrogen supplies in Yellow Sea waters, plus ability of the macroalgae to optionally use C4 photosynthetic pathways that facilitate rapid growth. Stable isotopic evidence shows that the high nitrogen supply is derived from anthropogenic sources, conveyed from watersheds via river discharges, and by direct atmospheric deposition. Wastewater and manures supply about half the nitrogen used by the macroalgae, fertiliser and atmospheric deposition each furnish about a quarter of the nitrogen in macroalgae. The massive green tides affecting the Yellow Sea are likely to increase, with significant current and future environmental and human consequences. Addressing these changing trajectories will demand concerted investment in new basic and applied research as the basis for developing management policies.

## Introduction

**Figure 1:**
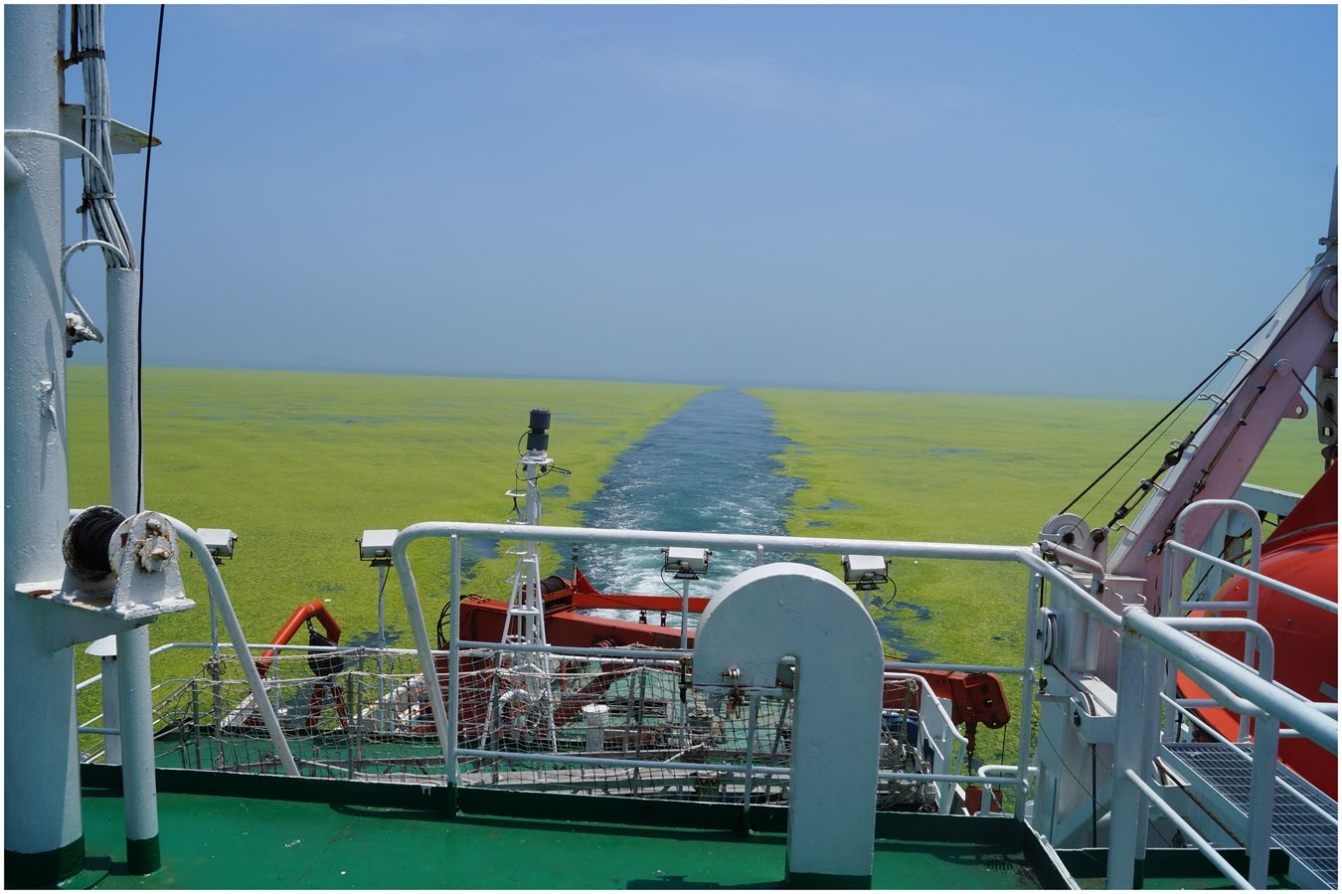
Shipboard view of the Yellow Sea green tide canopy of floating macroalgae. Photo by Dongyan Liu.

**Figure 2:**
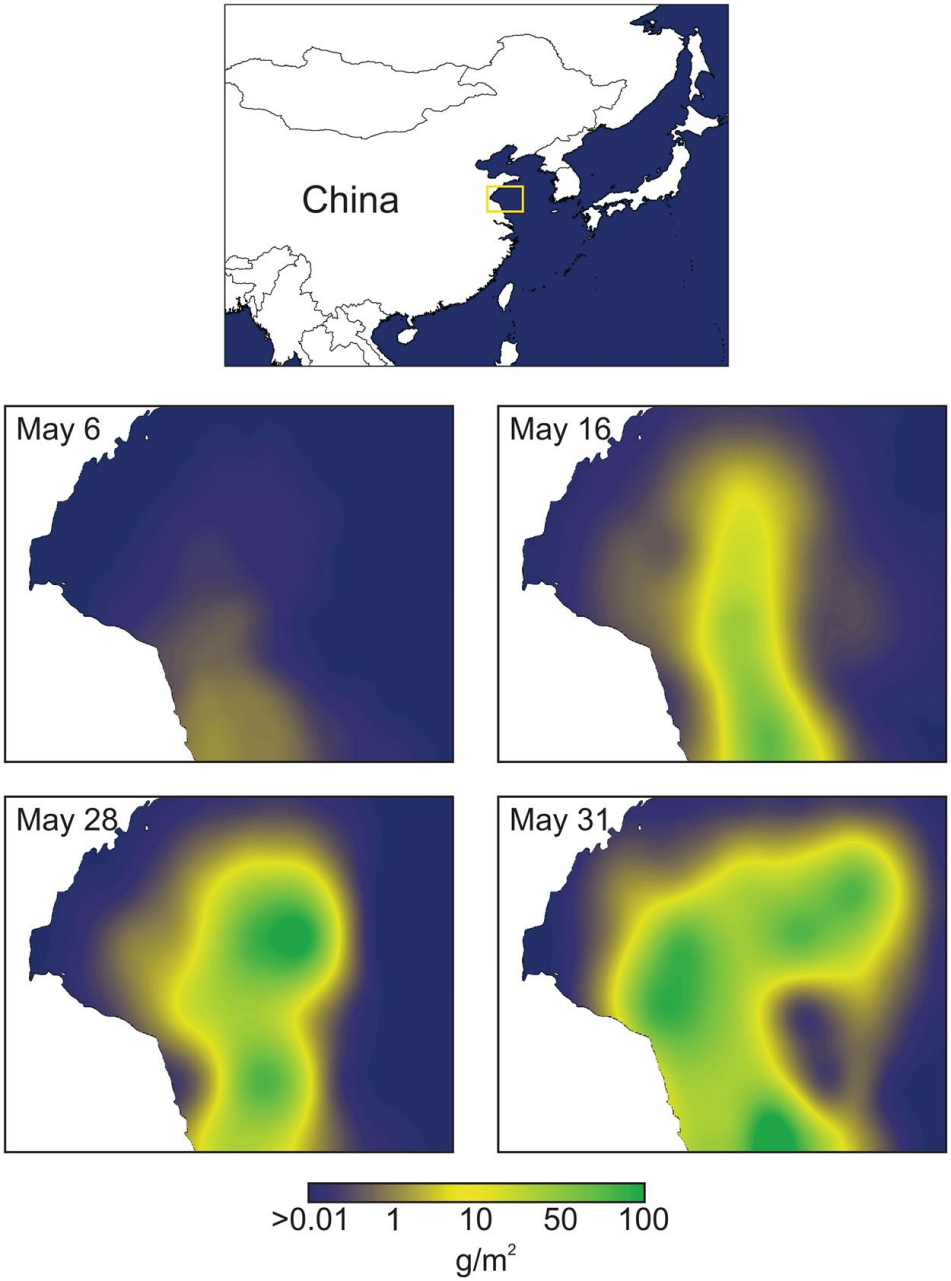
Top panel: location of the green tide sampling area on the Yellow Sea. Bottom four panels show the rapid expansion of the green tide between May 6 and May 31, done by GIS kriging of data^8^.

In mid-2008, press reports noted that the sailing competition of the XXIX Olympiad in Qingdao, China, were threatened by the presence of a massive canopy of green seaweeds floating on the race course^1,2^. These reports were followed by impressive images of accumulations of algal biomass on shore, which required activation of about 10,000 people, including the military, to clean up and bury. The stranding of masses of algae has become an annual event to be suffered locally, and press coverage has continued^3^. The algae involved have been identified^4,5^, and the processes underlying the annual blooms described^6,7^. Propagules of *Ulva prolifera*, the main species involved, first grow near-shore, attached on maricultural rafts, which are dislodged during harvest and cleaning activities in spring, and the fragments become propagules that are then free to float offshore and grow across a large portion of the Yellow Sea (Fig. 1). Even though some of the floating algae drift unto shore to create the massive strandings, the macroalgae proliferate in surface waters of the Yellow Sea and the macroalgal canopy expands rapidly (Fig. 2). *U. prolifera* grow remarkably quickly: in less than one month during 2012, floating canopies expanded up to 36,450 km^2^ across the Yellow Sea (Fig. 2), with a 62-fold increase of biomass^8^. In 2009, 2014, and 2015, the maximum area of the green tide reached 50,000–60,000 km^2^. For the sake of comparison, these green tides expand to areas that are three times the area of Wales, or almost half the size of England, or enough to cover the entire surface of Lake Michigan. The remarkably fast growth (>23% day^−1^)^9^ implicit in such a rapid expansion of the algal canopy calls for some explanation.

The expansive canopies of macroalgae that grow across the Yellow Sea have become known as the largest coastal green tides in the world. They have attracted their share of attention because of the cost of dealing with the annual green tides, the nuisance aspects on shore, disruption to mariculture, and because of scientific interest in the unusual phenomenon^10^. There have been many recent publications^6,7^, with much of the focus on the causes and controls of green tides.

Nitrogen supply is a major control of growth of coastal macroalgae such as *Ulva*^11,12^, so it is not surprising that the onset of growth of the green tides on the coast of the Yellow Sea depends on the large concentrations of available inorganic and organic nitrogen^13,14^, plus suitably warm temperatures^8^.

Concentrations of ammonium and nitrate in coastal waters surrounding China, and the Yellow Sea region in particular, have increased dramatically during recent decades^15–17^; for example, mean nitrate concentrations increased 7-fold between 1985 and 2010^17^. Concentrations of ammonium and nitrate discharged by rivers into the Yellow Sea are quite high (Table 1). Even in the open Yellow Sea, concentrations of dissolved inorganic nitrogen are 10–80 µM near the coast, and 0.7–5.8 µM offshore^7^. Other reports confirm that concentration of coastal dissolved inorganic nitrogen range 7.4–95 µM^18^ and 1–15 µM offshore^10^. Such high concentrations of nitrogen must stimulate the onset and maintain the *Ulva* bloom. N/P values are high, ranging from 48/1 to 259/1. These values are considerably above the 16:1 Redfield ratio, suggesting that there is sufficient nitrogen such that development of green tides in the Yellow Sea later in the season may be limited by phosphorus supply^10,14,17,19^, or not be nutrient-limited at all^20^.

**Table 1:** Range of concentrations of nitrate and ammonium as well as δ^15^N in NO_3_, in several Chinese river discharges into receiving seas.

Rivers	Range of concentrations (µM)		δ^15^N in NO_3_ (‰)	Source
	NO_3_	NH_4_		
Changjiang (Yangtze)	10–160	—	−4.6 to 8.9	^15^
“	20–140	—	—	^16^
“	76–152	—	2.3 to 3.8	^77^
“	90–120	—	—	^8^
“	44.8–109.3	0.1–39.9	—	^78^
“	—	0.25–25	—	^23^
“	27.1	0.5	12.8	^66^
Huanghe (Bohai Sea)	200–260	1.7–4	—	^79^
“	—	0.2–9	—	^23^
“	21.6–530	7.9–18,786	—	^80^
Jiulong (East China Sea)	14.3–743	0.2–1036	2.5–27	^81^
“	10–235	0–410	—	^82^
Pearl River (South China Sea)	0–325	0–850	—	^83^

**Figure 3:**
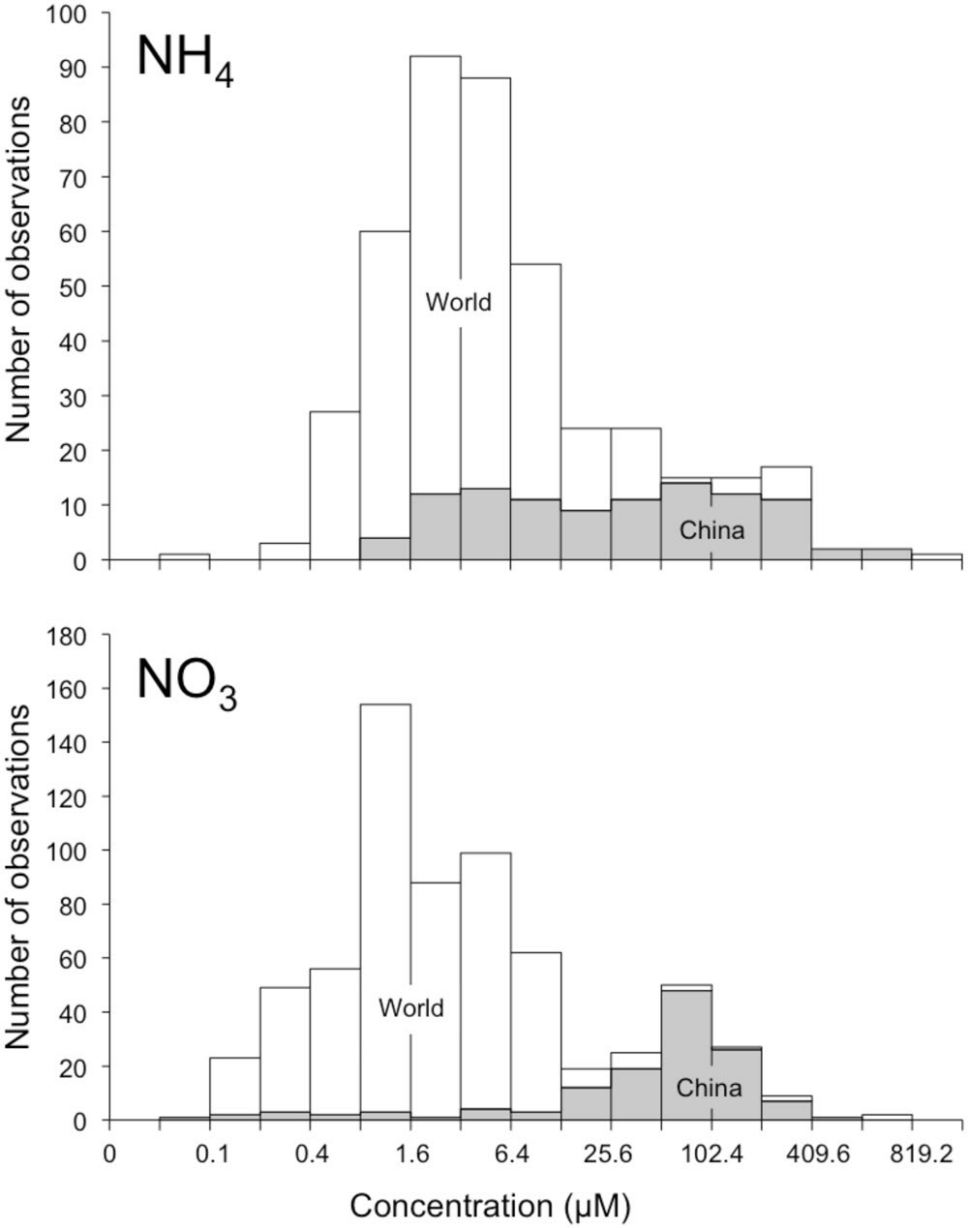
Frequency distributions of ammonium and nitrate in coastal Chinese waters and in coastal seawater from many stations around the world. Data compiled from three sources^73–75^.

The alarming rise of eutrophication of Chinese coastal waters follows from remarkable increases in nitrogen loads, transported by rivers, and by direct atmospheric deposition. Increasingly, watersheds discharge nitrogen from wastewater disposal, fertiliser and manure use, and atmospheric deposition on land, into rivers^17,21–25^. The increased concentrations and loads borne by rivers then translate into increased discharges to the Yellow Sea. For instance, discharges of nitrogen from the Yangtze River increased by 135% between 1980 and 2010^26^. Direct atmospheric deposition on coastal waters has also increased^19,27,28^, and may be involved in a tripling of the nitrate concentration in the Yellow Sea West of Korea^19^. Such recent increases in fluvial and atmospheric contributions have skewed coastal nutrient concentrations in Chinese coastal waters toward larger values than those found in seawater across other coastal regions of the world, particularly in the case of nitrate (Fig. 3).

The literature^7,19,25,29–34^ reflects broad agreement that, in general, in the Yellow Sea and its watersheds, human activities have increased atmospheric nitrogen deposition, that applications of agricultural fertiliser are excessive (more than half of the added fertiliser nitrogen is not used by crops, and is released into the environment), that rivers transport nitrogen from human and animal wastes, fertiliser use, and atmospheric deposition on watersheds to coastal waters, that direct atmospheric deposition on the Yellow Sea is significant, and that natural biological sources, such as fixation of nitrogen within the coastal environment, are much less than anthropogenic contributions. There is also little doubt that human and animal waste materials, fertilisers, and atmospheric nitrogen deposition, all support the remarkable green tides of the Yellow Sea.

**Figure 4:**
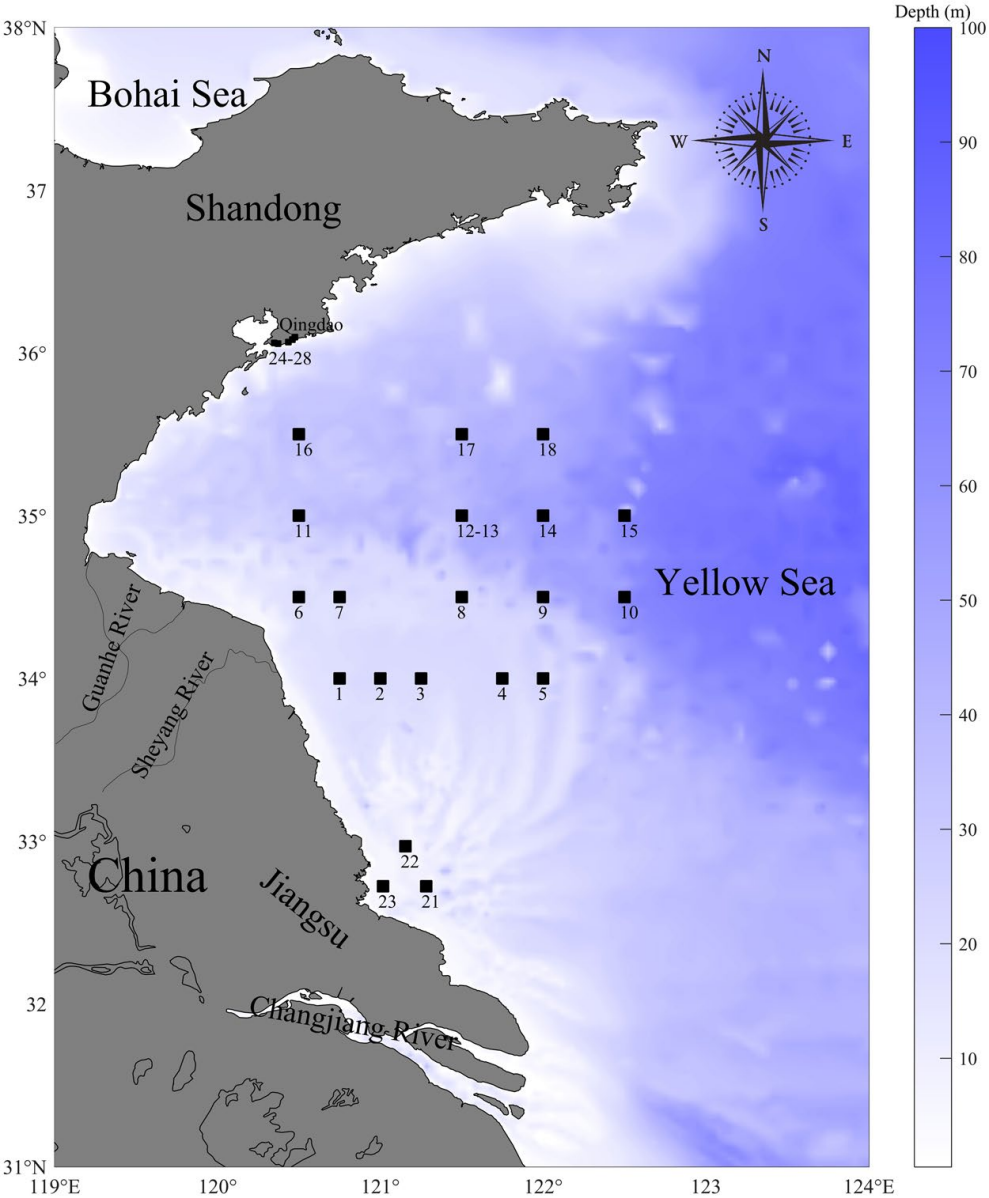
Map and location of sampling stations within the Yellow Sea where the green tide macroalgae were collected.

In contrast, there are substantial disparities in published estimates of magnitudes of contributions by different sources to nitrogen budgets of Chinese coastal waters. For example, some references conclude that rivers contribute 52% of nitrogen inputs to the Yellow Sea, while direct atmospheric deposition on the sea surface adds 42%^35^. Other references provide yet more differing estimates of atmospheric deposition to the Bohai Sea (Fig. 4) but focus on high deposition of NH_4_ and lower deposition of NO_3_^36,37^. Others argue that river discharge of nitrogen is much smaller than atmospheric deposition^23^, while still others aver that rivers may carry perhaps 62% of nitrogen loading into the Yellow Sea, with direct atmospheric deposition adding about 36%, and mariculture adding only about 2%^17^. The latter is much smaller than an estimate that mariculture activity may be of the same magnitude as atmospheric deposition^27^. Others conclude that increasing discharges of animal manure to rivers is the major cause of eutrophication^24^. Such disparities among published assessments of relative magnitude of different terms emerge from differences in processes and inputs considered, the forms of nitrogen included, and contrasts in area, estuary, or region studied. Calculated magnitudes of contributions by different sources differ enough to challenge comprehensive synthesis of nitrogen budgets, and confusing interpretations as to the relative importance of the drivers governing the green tide phenomenon.

We add, in passing, that nitrogen load estimates for the region largely omit consideration of two features that have been found to be important elsewhere. First, contributions via groundwater flow for Chinese coastal sites are significant^38,39^, and may reach nutrient discharges equivalent to river fluxes^40^. Second, inputs and dynamics of dissolved organic nitrogen are large for Chinese river discharges^9,41^. Both of these aspects merit more attention, as they are likely to be of a quantitative magnitude that changes perspectives on nutrient loading and transformations.

While it is difficult to make mass flow comparisons based on the published data, stable isotope analyses can furnish an empirical check on the relative magnitude of contributions from the various sources of nitrogen. Measuring stable isotopic values of macroalgae has been widely used to partition such contributions^42–46^.

In this paper, we first use stable carbon isotopic signatures of the macroalgae to see if they furnish insight into the remarkably fast growth of macroalgae in the Yellow Sea. The carbon isotope signatures were also used to observe the relationship between C3 and C4 metabolic pathways of the macroalgae. Stable isotopic nitrogen signatures of macroalgae collected from a series of Yellow Sea stations during a green tide event (Fig. 4) were examined to ascertain the degree to which different sources (human and animal wastes, fertilisers, and atmospheric deposition) are responsible for a high supply of available nitrogen, and hence for the green tides in the Yellow Sea.

## Results and Discussion

### δ^13^C values in Yellow Sea macroalgae

**Figure 5:**
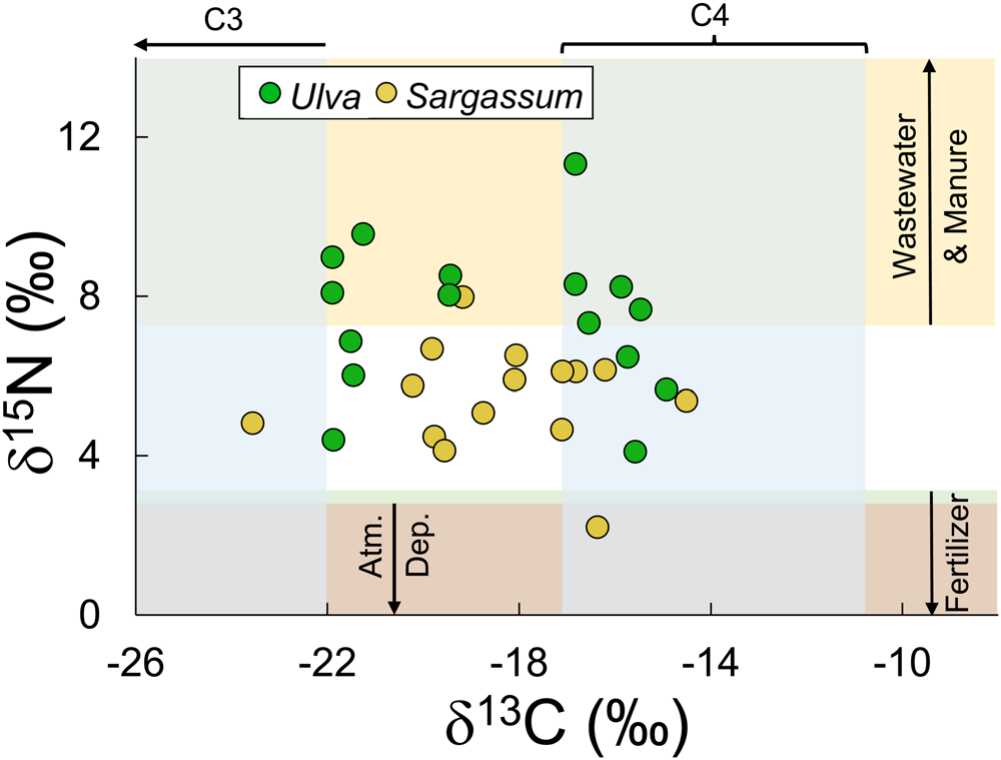
δ^15^N and δ^13^C of *Ulva prolifera* and *Sargassum horneri* from samples collected in stations shown in Fig. 4. For comparison, we added range of δ^13^C values for algae with C3 and C4 photosynthesis (along the x axis)^76^, and ranges of values for δ^15^N derived from human wastewater and livestock manure fertilisers, and atmospheric deposition (stable isotopic range data from sources in Table 4).

Measurements of δ^13^C in the samples of macroalgae collected in the stations of Fig. 4 ranged from −24 to −15‰ (x axis in Fig. 5). This is an unusual δ^13^C range for macroalgae, but similar values have been reported by others (Table 2). In general, producers—plants and algae—found in coastal aquatic environments carry out carbon fixation via C4 or the C3 metabolic pathways^47^. These pathways are characterised by differences in biochemistry and architecture^48^, particularly internal air spaces where CO_2_ and carbonate may be re-used. Presence of C3 or C4 metabolism is associated with relatively constrained values of δ^13^C (Table 2). Most algae use the C3 pathways for fixing carbon and have corresponding δ^13^C values; the contrasting range of δ^13^C found in *Ulva* in the Yellow Sea and elsewhere (Table 2) is therefore in need of explanation.

**Table 2:** δ^13^C (mean ± se) in C3 and C4 producers, and ranges of δ^13^C in fronds of selected macroalgal species collected from different locations.

	δ^13^C (‰)	Location	Source
C3 producers	−28.1 ± 2	—	^84^
C4 producers	−13 ± 1.5	—	“
C3 producers	−22 to −35	—	^76^
C4 producers	−11 to −17	—	“
**Green algae:**			
*U. prolifera*	−21.9 to −14.9	Yellow Sea	This study
*Ulva* sp.	−14.2 to −10.1	New South Wales coast	^85^
*Ulva lactuca*	−13 to −11	SE Brazil coast	^86^
“	−22.5 to −22.3	Mediterranean Sea	^87^
*Ulva intestinalis*	−12.5	Neva estuary	^88^
*Cladophora glomerata*	−15.5 to −12.5	“	“
*Cladophora* sp.	−20 to −18	Gulf of Mexico	^89^
*Codium* sp.	−15 to −14	SE Brazil coast	^86^
*Caulerpa prolifera*	−14.1 to −13.8	SE Iberian coast	^90^
**Brown algae:**			
*Sargassum horneri*	−23.6 to −14.5	Yellow Sea	This study
*Sargassum natans*	−20 to −18	Gulf of Mexico	^89^
*Sargassum fluitans*	−17 to −16	“	“
*Sargassum* sp.	−19.5 to −16	“	^91^
*Sargassum* sp.	−16.3 to −10	New South Wales coast	^85^
*Sargassum vulgare*	−13 to −12	SE Brazil coast	^86^
*Sargassum* sp.	−15 to −12.5	Baltic Sea	^88^
*Cystoseira amentacea*	−19.7 to −15.8	Mediterranean Sea	^87^
**Red algae:**			
*Pterocladium capillacea*	−16.7 to −13.5	New South Wales coast	^85^
*Gelidiella acerosa*	−19	SE Brazil coast	^86^
*Pterocladiella capillacea*	−33 to −32	“	“
*Cryptonemia seminervis*	−30	“	“
*Gracilaria* sp.	−16.5 to −15	“	“
*Hypnea musciformis*	−17.5 to −16	“	“
Compilation of 10 macroalgal species	−21.7 to −11.3	Coast of Cape Cod	^92^
Compilation of 11 macroalgal species	−20.9 to −10.5	Caribbean Sea	^93^

It turns out that C3 and C4 photosynthetic pathways co-occur in *Ulva*, as demonstrated by transcriptome sequencing that revealed presence of C4 and C3 genes, as well as shown by presence and activity of enzymes involved in C4 metabolism^49,50^. These results confirm earlier findings that C4 and C3 characteristics co-occur in certain producers^51^, and that C4 metabolism evolved in different plant groups, and at different geological periods^52^, rather than exclusively in grasses in the late Miocene. The aspects of C4 metabolism most relevant here is that this pathway minimises photorespiration, increases photosynthetic efficiency, raises nutrient uptake efficiency, and favours high rates of photosynthesis—even where CO_2_ concentrations are low^53,54^. These features potentially confer high rates of growth and productivity, which can in turn lead to the ability to accumulate biomass at much faster rates than possible with C3 pathways. It has been speculated that the remarkable fast development of green tides in the Yellow Sea may be supported by sustained release of propagules from maricultural rafts^55^ and high supply of nutrients^14,17^. We conjecture that perhaps the fast growth associated with ability to carry out C4 metabolism—revealed by the isotopic evidence in Fig. 5— might be an additional and important explanation, particularly adapted for fast growth in waters where there is a high supply of available nitrogen.

It seems likely that macroalgae other than *Ulva* might be able to carry out combinations of C3 and C4 metabolism. The brown macroalga *Sargassum* showed δ^13^C values ranging −24 to −14‰ (Fig. 5), and there are many reports of ranges of δ^13^C that span values between those typical of C3 and C4 metabolism. These results suggest that the co-occurrence of C3 and C4 carbon fixation pathways might be widespread among macroalgae (Table 2).

There is a further implication of the confirmation that C4 and C3 metabolism co-occur in certain macroalgae. As atmospheric CO_2_ rises in coming decades, more CO_2_ will be stored in the oceans. If nutrient supply increases, macroalgae metabolically pre-adapted to efficiently fix carbon seem therefore likely to proliferate, much as they have done in the Yellow Sea, across other nutrient- and carbon-enriched coastal waters of the world^56,57^. A number of potential biogeochemical and ecological consequences might ensue. There are also concerns about the potential for green tides to foster wholesale shifts in the composition of producers (and of food webs) in the water column^58^. Competition for nutrients with other producers, such as phytoplankton, seems implausible, as concentrations of dissolved inorganic nitrogen remain high in the Yellow Sea through the growing season. It seems more likely that in the Yellow Sea competition with phytoplankton might be mediated by shading by macroalgal canopies. There may be increased delivery of carbon to deeper layers of the sea as green tides senesce and biomass sinks. The shifts in metabolism in the producers, associated with high nitrogen supply, might therefore extend to the alter food webs in the Yellow Sea and other similarly affected ecosystems. Testing of such possibilities will be of interest.

### δ^15^N values in Yellow Sea macroalgae

The values of δ^15^N in samples of the green macroalga (*U. prolifera*) taken from the stations in Fig. 4 span a range of 4 to 11‰ (y axis in Fig. 5). The range we measured in *Ulva* reasonably overlap values in other reports, for green, brown, and red macroalgae (Table 3).

**Table 3:** Ranges of δ^15^N in selected macroalgal taxa collected from different sites.

	δ^15^N (‰)	Site	Sources
**Green algae:**			
*U. prolifera*	4.1 to 11.3	Yellow Sea	This study
“	3.2 to 10.1	“	^18^
“	3.9 to 23.3	“	^10^
*Ulva lactuca*	5.7 to 8.2	Mediterranean	^87^
“	7.4 to 13.6	“	^94^
“	8 to 8.3	SE Brazil coast	^86^
*Ulva intestinalis*	8.5	Neva estuary	^88^
*Cladophora glomerulata*	6	“	“
*Cladophora* sp.	8.5 to 10	Gulf of Mexico	^89^
*Codium* sp.	9.2 to 9.6	SE Brazil coast	^86^
*Caulerpa prolifera*	2.7 to 8.6	SE Iberian coast	^90^
**Brown algae:**			
*Sargassum horneri*	2.2 to 8	Yellow Sea	This study
*Sargassum natans*	1.4 to 3.5	Gulf of Mexico	^89^
*Sargassum fluitans*	2.3 to 3.1	“	“
*Sargassum* sp.	−1.5 to 1.5	“	^91^
*Sargassum vulgare*	8 to 8.4	SE Brazil coast	^86^
*Sargassum* sp.		Baltic Sea	^88^
*Cystoseira amentacea*	7.1 to 8.2	Mediterranean	^87^
*Ascophyllum nodosum*	5.1 to 10.1	NW Iberian coast	^95^
*Fucus vesiculosus*	1.6 to 13.8	“	“
**Red algae:**			
*Gracilaria* sp.	6.6 to 7.3	SE Brazil	^86^
*Gelidiella acerosa*	6.6 to 7.6	“	“
*Pterocladiella capillacea*	5.4 to 6.2	“	“
*Cryptonemia seminervis*	6.4 to 7.1	“	“
*Hypnea musciformis*	8.4 to 9.4	“	“
Compilation of many macroalgal taxa:			
Greens:	1.9 to 12.8	Coast of Vietnam	^96^
Browns:	2.3 to 7.1	“	“
Reds:	1.9 to 10.8	“	“
Compilation of many macroalgal taxa:			
	4 to 18	Coasts of the world	^97^

Many of the entries in this table are based on means, hence the ranges are underestimates. One of the estimates from ref.^18^ had an anomalous range of −6.7 to −1.3‰ and was not included in this entry.

The δ^15^N values we measured in the Yellow Sea *U. prolifera* reflect uptake of inputs of nitrogen entering these coastal waters, plus within-estuary biochemical nitrogen cycle transformations. For the Yellow Sea, the nitrogen inputs are, to a degree, better known than the internal biogeochemical transformations. Here we therefore focus on the nitrogen inputs. To interpret the distribution of points in Fig. 5, we compare the position of measurements from the samples in relation to reasonably well-established bounds reported for stable isotopic values on nitrogen in nitrate (Table 4). These bounds are shown in Fig. 5.

**Table 4:** Ranges and midpoints of δ^15^N values in inorganic sewage and manures, fertilisers, atmospheric deposition, and soils.

Nitrogen sources	Ranges of δ^15^N (‰)	Midpoint of δ^15^N range (‰)
Human sewage and animal manures	7.3 to 21	14.2
Inorganic fertilisers	−3.9 to 3.1	−0.4
Atmospheric deposition	−8.1 to −2.9	−5.5
Soils	0.3 to 7.4	3.9

Values averaged from compilations in the literature^44,70,80,98–107^. These compilations included some of the same sources of information. To quantify inorganic fertilisers, as available, we mainly used isotopic values for ammonium-based fertilisers, because Chinese farmers use primarily ammonium and urea rather than nitrate fertilisers^29^, and urea released into aquatic environments is rapidly hydrolysed to ammonium. The ranges of isotopic signature for human waste and animal manures overlap so closely that we combined both into a single range. It would be of interest to find ways to separate these two sources, since mass balance estimates of the relative magnitude of these sources do not agree^24,25,29^. The soils and sediments transported by rivers are not a clear example of an input, since they carry nitrogen that was delivered by atmospheric deposition, fertilisers, wastewater and manures, and other inputs. This item is included here to show its δ^15^N range is intermediate, as befits a bearer of a mix of nitrogen from different sources.

We highlight major *external* anthropogenic sources of N, including human wastewater and animal manures (the latter is likely a smaller contribution, since in our unpublished review of fate of manure N in watersheds, we found that only 3.7% of manure N reaches receiving coastal waters), fertilisers, and atmospheric deposition.

Other sources of nitrogen, which are likely to make lesser contributions to the Yellow Sea, include river-transport of soils and sediments, nitrogen fixation, inputs from the extensive mariculture industry in the region, and contributions of “natural” nitrogen from upwelled deeper water or wandering Kuroshio Stream sources. The nitrogen brought into the Yellow Sea by sediments and soils holds a mix of what was introduced by use of fertilisers, disposal of wastes, and by atmospheric deposition, all on watersheds. The intermediate values of the δ^15^N for soils (Table 4) reflect that mix of sources. To avoid possible double-accounting of these sources, we did not consider nitrogen in soil particles brought into the sea by river transport. There is evidence of some nitrogen fixation from the detection of diazotrophic microorganisms in the Yellow Sea water column^59^. In earlier studies, nitrogen fixation only amounted to 6% of the nitrogen inputs to the Yellow Sea. Since then, available ammonium has increased in the water column, which should further depress the contribution by nitrogen fixation^60^, and hence, we ignored fixation here. Nitrogen inputs from mariculture activities need to be considered in the context that there is a countering removal of nitrogen inherent in the industrial-level macroalgal culture in the Yellow Sea^17,27,61^. For the Chinese coast as a whole, by 2010, shell- and fin-fish culture may have released 0.2 × 10^6^ tons of nitrogen per year^61^. The nitrogen removed in harvests of macroalgal crops reached 2 × 10^6^ tons in 2014^60^. Clearly, maricultural efforts are a net remover of nitrogen from the Yellow Sea and might be an effective management counter to increased nitrogen loadings^62–64^. In regard to upwelled N sources, we found no measurements of δ^15^N in deep-layer nitrate within the region, but it has been reported that δ^15^N of particulate organic matter (POM) from deeper layers ranged from 3.1 to 5.8‰^65^, which should be somewhat heavier than those of the nitrate taken up by the POM. δ^15^N signatures of nitrate upwelled from deeper layers should therefore be lighter than those of nitrate derived from wastewater discharged from watersheds into the sea. Possible nitrate inputs from Kuroshio wanderings have been discussed^66^, but river flow seemed to be the dominant source of nitrate in the localities where the green tide bloom started during the growing season (Fig. 2). The overwhelming dominance of fluvial N transport to the Yellow Sea is corroborated by many papers^18,67–71^. Moreover, during warmer seasons in the Yellow Sea, vertical stratification is marked, as evident in sigma t and salinity profiles, constraining upwelling of deeper layers so that upward transport of heavy N may not be a dominant mechanism^66^. In addition, the macroalgae discussed here float in the upper half meter of the water column or so. This is also the layer most affected by river flow, with lowest salinities. It is therefore not at all surprising that isotopic signatures of the floating green tides reflect fluvial inputs rather than inputs from deeper Yellow Sea layers or Kuroshio sources.

The distribution of δ^15^N values measured in samples of macroalgae from the Yellow Sea range from about 2 to 11‰ (Fig. 5). For both *U. prolifera* and *S. horneri*, the range of isotopic values reasonably match values of δ^15^N of nitrate contributed by rivers (Table 1), which seems reasonable because fractionation during uptake by algae is minimal, perhaps adding only 1‰ to the signature of the source. The lower isotopic values appear to be a result of uptake of nitrogen originally from fertiliser use and atmospheric deposition. The upper ranges of isotopic values in the macroalgae were considerably higher than would be expected if nitrogen from fertiliser and atmospheric deposition had been the main sources. In fact, most points in Fig. 5 fall in an intermediate region between those found in human and animal waste, and those characteristic of fertiliser and atmospheric deposition values (Table 4). This implies that a mix of these nitrogen sources was taken up by the macroalgae.

**Figure 6:**
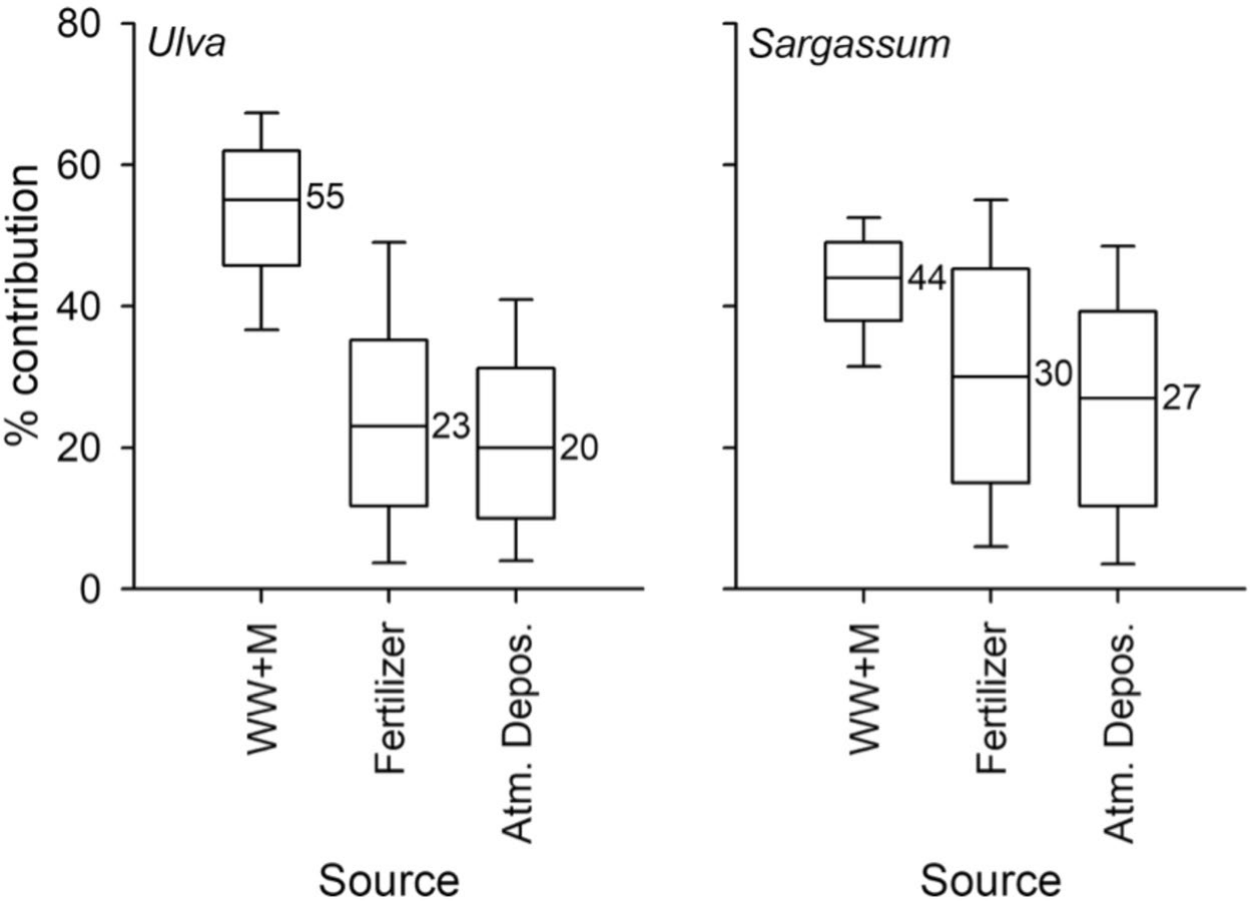
Percent contribution of the nitrogen measured in *U. prolifera* and *S. horneri* that were likely supplied by fertilisers, human wastewater plus livestock manure (WW + M), and by atmospheric nitrogen deposition. Calculations were done by the IsoSource stable isotope mixing model. Numbers are medians, boxes represent the central 50% of the distribution, and whiskers represent maximum and minimum values. Outliers not included.

To obtain approximate estimates of the relative contributions from the most likely and distinguishable sources, (1) human and animal wastes; (2) fertilisers; or (3) atmospheric deposition, we used IsoSource, a stable isotope mixing model^72^. To simplify the calculation, we entered the mid-point in the range of δ^15^N for each of the three sources (Table 4) and calculated the % contribution of nitrogen in *Ulva* and *Sargassum* for the samples included in Fig. 5. The values for wastewater (and to a rather smaller extent, manures) differ clearly from those in fertiliser and atmospheric deposition. The latter two sources bear similar signatures, which impairs partition. The salient result from the IsoSource partition, however, is that about half the N taken up by both species of macroalgae derived from anthropogenic wastewater in the Yellow Sea as a whole. Fertilisers and atmospheric deposition each may have added a quarter of the N found in the macroalgae (Fig. 6). The proportions of waste N uptake were larger (about 60%) in the more coastal regions of Qingdao and Subei (Fig. 4), perhaps suggesting that near-shore environments are more subject to waste disposal effects.

The substantial degree of eutrophication in Chinese rivers and coastal water, made strikingly evident by the massive green tides, suggests two high priorities. First, developing management policies to address the issues will need greater understanding of basic aspects. While we show above that certain sources of nitrogen seem important, examination of the literature shows that actual estimates of mass fluxes from different sources, and of flows though rivers, groundwater, and atmospheric deposition differ from one publication to another. Concerted and critical synthesis of published and new work is needed to constrain the estimates into a comprehensive context. Better quantified knowledge of the sources, and transport routes, will go a long way to suggest how to best target approaches to manage nitrogen loads. Second, while work on developing approaches to manage N loads is taking place, it seems also important to assess the various basic and applied effects of the green tides. One obvious aspect is to understand the economic, social, and infrastructural costs; another might be to develop work aiming at understanding the environmental effects on the Yellow Sea ecosystem. These might include major current and future changes in biodiversity, food webs, economically important shell and fin-fish stocks, and biogeochemistry, exerted by the remarkable re-routing of nutrient fluxes, carbon production and sinking, shading, and most likely other still-unknown changes.

## Methods

Field surveys from a small craft were carried out during May-June 2017, sampling macroalgal biomass at a series of stations in Jiangsu and Qingdao coastal waters, and aboard RV Su88 across the western Yellow Sea, covering a region of 32.67–33.67°N and 120.50–122.70°E (Fig. 4). Samples of the macroalgae collected were dominated by a green (*Ulva prolifera*) and a brown (*Sargassum horneri*) species. Macroalgal samples were briefly rinsed in filtered seawater and frozen.

The frozen macroalgal samples were dried in a Christ ALPHA 1–4 LSC freeze dryer and ground in the East China Normal University laboratory in Shanghai. Samples of ground macroalgae were then shipped to the Stable Isotope Laboratory at the Marine Biological Laboratory in Woods Hole, MA to be analysed for carbon and nitrogen content, as well as carbon, nitrogen, and sulphur stable isotope signatures. The stable isotopic values were determined using a Europa 20–20 Continuous-Flow Isotope Ratio Mass Spectrometer system. All data analysed during this study are included in this article. Any further inquiries can be directed to the authors.
